# Tobacco Smoking among the Online Respondent Dental Students of a Dental College: A Descriptive Cross-sectional Study

**DOI:** 10.31729/jnma.7503

**Published:** 2023-03-31

**Authors:** Deepak Thakur, Navpreet Kaur, Monika Chahar, Nitul Barman, Roopali Gupta, Vivek Sharma

**Affiliations:** 1 Department of Public Health Dentistry, K.D. Dental College and Hospital, Mathura, Uttar Pradesh, India

**Keywords:** *dental students*, *smoking*, *tobacco cessation*

## Abstract

**Introduction:**

Smoking tobacco is most common nowadays among dental students due to stress induced by practical workloads and exams. There is limited data regarding tobacco smoking among dental students. The aim of this study was to determine the prevalence of tobacco smoking among online respondent dental students of a dental college.

**Methods:**

This was a descriptive cross-sectional study conducted on dental students from 15 July 2021 to 15 August 2021. Ethical approval was obtained from the Institutional Review Committee of K.D. Dental College and Hospital (Reference number: KDDC/Admin/2021/9990A). Data was collected through a structured questionnaire and responses were gathered using an online Google form survey with informed consent. A convenience sampling method was used. Point estimate and 95% confidence interval were calculated.

**Results:**

Among 60 online respondents, the prevalence of tobacco smoking was found to be 11 (18.33%) (17.04-24.56, 95% Confidence Interval). The percentage of participants who wanted to stop smoking now was 11 (18.33)%.

**Conclusions:**

The prevalence of tobacco smoking among the online dental respondents of a dental college was similar to the other studies done in similar settings.

## INTRODUCTION

According to the World Health Organization (WHO), tobacco kills more than eight million people each year; seven million deaths due to tobacco use and 1.2 million due to passive smoking.^1^ Nearly 90% of lung cancer deaths and 80% of deaths from Chronic Obstructive Pulmonary Disease (COPD) are due to tobacco smoking.^2^ Tobacco use has been shown to be the sixth of eight leading causes of death worldwide.^3^

An estimated 4.9 million deaths every year can be linked to smoking tobacco.^4^ Data was lacking which compares the condition of dental students regarding smoked tobacco. This survey can assist in prioritizing and developing tobacco control programs, including surveillance, evaluation, and policy development.^5^

The aim of this study was to find out the prevalence of tobacco smoking among online respondent dental students of a dental college.

## METHODS

This study was a descriptive cross-sectional study conducted in K.D. Dental College and Hospital students from 15 July 2021 to 15 August 2021 through a pre-validated structured questionnaire adopted from the Global Youth Tobacco Survey (GYTS)^6^ after obtaining ethical approval from K.D. Dental College and Hospital, Mathura (Reference number: KDDC/Admin/2021/9990A). Sample size calculation was done by using the following formula:


$$\begin{array}{ll} \text{n} & ={\text{Z}}^{2}\times \frac{\text{p}\times \text{q}}{{\text{e}}^{\text{2}}}\\ & =1.96^{2}\times \frac{0.17\times 0.83}{0.10^{2}}\\ & =55 \end{array}$$

Where,

Z = 1.96 at 95% Confidence Interval (CI)
p = prevalence of smoking taken from previous studies, 17%^7^
q = 1-p
e = margin of error, 10%

Thus, the calculated minimum required sample size was 55. However, 60 patients were taken for the study.

The responses were gathered using an online Google form survey by convenience sampling method. Standard procedures of informed consent were used, including the protection of participant anonymity and confidentiality. The dental students from K.D. Dental College and Hospital were included in the study. The proforma consisted of demographic details such as name, age, gender, dental college, and a questionnaire regarding smoked tobacco. All the dental students participated in a survey on a voluntary basis. All the dental students from K.D. Dental colleges were given online Google forms and those who were voluntarily willing to participate in the survey were included. An adult who had smoked 100 cigarettes in his or her lifetime and who currently smokes cigarettes were defined as a smoker.^8^ Those dental students who did not respond were excluded from the study. The collected data were entered into and analysed using Microsoft Excel version 16. Point estimate and 95% CI were calculated.

## RESULTS

Among 60 online respondents, the prevalence of tobacco smoking was found to be 11 (18.33%) (17.0424.56, 95% CI). (Table 1).

**Table 1:** Online responses of dental regarding tobacco smoking variables students (n = 11).

Tobacco smoking variables	n (%)
Smoking at 14 or 15 years old	1 (9.09)
Smoking at 16 years old or older	10 (90.11)
Think of using any form of tobacco during next 12 months	8 (72.72)
Not Think of using any form of tobacco during next 12 months	3 (27.28)
Received help from program or professional to stop smoking	1 (9.09)
Received help from friends to stop smoking	4 (36.36)
Received help from family members to stop smoking	2 (18.18)
Received help from both programs or professional and friends or family members	4 (36.36)
Think that they would be able to stop smoking if they wanted to	2 (18.18)
Think they would not be able to stop smoking	9(81.81)
Agreed that there is need of more information on tobacco cessation methods in undergraduate curriculum	6 (54.54)
Disagreed that there is need of more information on tobacco cessation methods in the undergraduate curriculum.	5 (45.45)

## DISCUSSION

In our study prevalence rate of tobacco smoking was 18.33% among dental students which was similar to one of the studies in which the prevalence was found to be 17%.^7^ Similar finds were found in other studies.^9^ As dental students have to face continuous exams and practical workloads, many of them think that cigarette smoking helps them temporarily cope with stress and likewise, they begin smoking. Smoking tobacco among dental students and their chances of tobacco cessation is still not a topic that is widely discussed. In one of the study it is found that, the dental student have good knowledge about the effect of tobacco on health.^10^

In the present study, 10 (90.11%) college students smoked cigarettes at 16 years old or older which is similar to the study done in a similar setting.^7,11^ The average age of initiating smoking was 16 years or older which was similar to one of the studies.^7^ In the present study 11 (9.56%) students responded that they would want to quit smoking now which was similar to the study conducted.^12^

Most dental students responded that they had enough information during their studies to use tobacco cessation methods in their dental practice which was similar to the study conducted in a similar setting,^11^ and another similar study has the same finding.^11^ In the present study 6 (54.4%) dental college students responded that there was a need for more information on tobacco cessation methods in the undergraduate curriculum which was similar to the study.^7^

The limitation of the study is that the questionnaire did not differentiate cigarette smoking from e-cigarette smoking. The lower response rate might have affected the generalizability of the results. The smoking status of the participants was assessed through self-report, rendering this study result comparably less reliable. This study was conducted only in one dental college which is a homogeneous population and needed to be done on more dental students and colleges. The cross-sectional design of the study does not allow us to make any causal inferences.

## CONCLUSIONS

The prevalence of dental students from dental colleges who were smoking tobacco was very low as compared to similar studies done in similar settings. The majority of dental students from dental colleges were aware of tobacco smoking's ill effects and recommended more information on tobacco cessation methods in the undergraduate curriculum.
